# Modeling Health and Economic Outcomes of Eliminating Sex Disparities in Youth Physical Activity

**DOI:** 10.1001/jamanetworkopen.2024.46775

**Published:** 2024-11-25

**Authors:** Kosuke Tamura, Marie F. Martinez, Yangyang Deng, Jessie Heneghan, Colleen Weatherwax, Kavya Velmurugan, Kevin L. Chin, Breanna Rogers, Mohammad Moniruzzaman, Meredith Whitley, Sarah M. Bartsch, Kelly J. O’Shea, Alexis Dibbs, Sheryl Scannell, Bruce Y. Lee

**Affiliations:** 1 Socio-Spatial Determinants of Health Laboratory, Population and Community Health Sciences Branch, Division of Intramural Research, National Institute on Minority Health and Health Disparities, National Institutes of Health, Bethesda, Maryland; 2 Public Health Informatics, Computational, and Operations Research, CUNY Graduate School of Public Health and Health Policy, New York, New York; 3 Center for Advanced Technology and Communication in Health, CUNY Graduate School of Public Health and Health Policy, New York, New York; 4 Artificial Intelligence, Modeling, and Informatics, Nutrition Guidance and Systems Center, New York, New York; 5 Ruth S. Ammon College of Education and Health Sciences, Adelphi University, Garden City, New York; 6 Maties Sport Centre for Sport Leadership, Stellenbosch University, Stellenbosch, South Africa

## Abstract

**Importance:**

Sex disparities in physical activity (PA) and sports participation among US children and adolescents have been persistent. Quantifying the impact of reducing or eliminating these disparities may help determine how much to prioritize this problem and invest in interventions and policies to reduce them.

**Objective:**

To quantify what might happen if existing PA and sports participation disparities were reduced or eliminated between male and female children and adolescents.

**Design, Setting, and Participants:**

This simulation study used an agent-based model representing all children (aged 6 to 17 years) in the US, their PA and sports participation levels, and relevant physical and physiologic characteristics (eg, body mass index) as of 2023. Experiments conducted from April 5, 2024, to September 10, 2024, simulated what would happen during the lifetime of each cohort member if PA and sports participation levels for female participants were increased (to varying degrees) to match male participants in the same age group.

**Main Outcomes and Measures:**

Health outcomes, such as body mass index, incidence of weight-related conditions (eg, stroke, coronary heart disease, type 2 diabetes, and cancer), and economic outcomes (eg, direct medical costs and productivity losses).

**Results:**

This simulation study modeled 8 299 353 US children and adolescents (4 240 119 [51.1%] male and 4 059 234 [48.9%] female) aged 6 to 17 years. Eliminating PA sex disparities averted 28 061 (95% CI, 25 358-30 763) overweight and obesity cases per cohort by age 18 years, which in turn averted 4869 (95% CI, 4007-5732) weight-related disease cases during their lifetimes and resulted in recurring savings of $333.45 million (95% CI, $290.22 million to $376.68 million) in direct medical costs and $446.42 million (95% CI, $327.39 million to $565.44 million) in productivity losses (in 2024 US dollars) for every new cohort of 6- to 17-year-olds. Reducing PA disparities by 50% averted 9027 (95% CI, 6942-11 112) overweight and obesity cases. Eliminating sex disparities in sports participation averted 41 499 (95% CI, 37 874-45 125) cases of overweight and obesity and 8939 (95% CI, 8088-9790) weight-related disease cases during their lifetimes, generating recurring savings of $713.48 million (95% CI, $668.80 million to $758.16 million) in direct medical costs and $839.68 million (95% CI, $721.18 million to $958.18 million) in productivity losses.

**Conclusions and Relevance:**

In this simulation study of youth PA and sports participation, eliminating sex disparities could save millions of dollars for each new cohort of 6- to 17-year-olds, which could exceed the cost of programs and investments that could enable greater equity.

## Key Points

**Question:**

What are the estimated health and economic outcomes of reducing or eliminating sex disparities in youths’ physical activity and sports participation?

**Findings:**

In this simulation study using an agent-based model of more than 8 million US children aged 6 to 17 years, eliminating physical activity disparities averted 28 061 cases of overweight and obesity by 18 years of age and 4869 weight-related disease cases during their lifetimes, saving $780.00 million. Eliminating sports participation disparities averted 41 499 and 8939 cases of overweight and obesity and weight-related diseases, respectively, saving $1.55 billion.

**Meaning:**

These cost savings could exceed the cost of programs and investments to enable such equity.

## Teaser

This simulation study of an agent-based model representing all US children aged 6 to 17 years examines what might happen if sex disparities in physical activity and sports participation were reduced or eliminated.

## Introduction

Numerous studies have shown persistent disparities in physical activity (PA) and sports participation levels between male and female children and adolescents in the US.^1,2,3,4^ For example, a study using the 2011-2019 Youth Risk Behavior Survey showed that only 15% of female participants vs 31% of male participants in grades 9 to 11 met their aerobic guidelines.^1^ Another study measuring sedentary behavior showed that female adolescents spent 6.14 to 8.13 hours per day in sedentary behaviors compared with 6.0 to 7.91 hours per day for male adolescents.^5^ Another study found that, in 2019, only 54.1% of female participants vs 60.4% of male participants participated in sports.^1^ Furthermore, another study found that 43.1% of girls vs 34.5% of boys reported never playing sports, 36.5% of girls vs 45.6% of boys were engaged in sports, and 36% of girls vs 30% of boys dropped out of sports.^6^

It is unlikely that such disparities would be eliminated or significantly reduced without additional attention (eg, social support from parents,^7^ friends,^8,9,10^ school environments, and social environment^11,12^), investment (eg, funding for new programs or initiatives), and resources (eg, sports and fitness programs, training and instruction, and guidelines). Therefore, it would be helpful to quantify the health and economic impact of continuing sex disparities in youth PA and sports participation. To date, studies have quantified the health and economic impact of increasing overall youth PA and sports participation, such as one study that showed that increasing the percentage of 8- to 14-year-olds in the US to meet the Centers for Disease Control and Prevention’s PA recommendation could decrease childhood overweight and obesity cases by 4% and save $8.1 billion in direct medical costs.^13^ Another study showed how meeting the Healthy People 2030 target of getting 63.3% of 6- to 17-year-olds to participate in sports could decrease overweight and obesity prevalence by 3.37% and save $80.0 billion.^14^

However, increasing overall PA and sports participation is not the same as reducing existing disparities and could even worsen such disparities if certain sociodemographic groups are left behind. In addition, the strategies for reducing sex disparities may be different and focus more on granting more equitable access to existing resources. Although one study showed that eliminating socioeconomic status-based PA disparities among US 6- to 17-year-olds could save $15.6 billion,^15^ there is a dearth of such studies based on sex disparities. To fill this gap, we adapted and used an agent-based model of all US children and adolescents to simulate what might happen if sex disparities in PA and sports participation were reduced or eliminated.

## Methods

### Agent-Based Model Overview

Using our previously described Virtual Population for Obesity Prevention agent-based model (developed in Python),^13,14,15,16,17,18,19,20,21^ we represented children and adolescents aged 6 to 17 years (starting in 2023 and simulated for the remainder of their lifetimes), their PA and sports participation, and subsequent health outcomes. Each child or adolescent is represented by a computational agent with a set of sociodemographic characteristics (eg, age and sex) as well as clinical characteristics (eg, fat-free mass and fat mass) based on the 2017-2020 National Health and Nutrition Examination Survey (NHANES).^22^ Data on race and ethnicity were captured in the NHANES data but are not a key parameter in our current study and are therefore not included. The model (eFigure in Supplement 1) proceeds in 1-day timesteps until 18 years of age, and each agent consumes a daily amount of calories to maintain a constant body mass index percentile accounting for baseline PA levels.^23^ Each agent has an embedded age-, sex-, and weight-specific metabolic model that tracks caloric consumption and energy expenditure and translates caloric surplus or deficit into weight gain or loss daily.^24,25^ After 18 years of age, the model proceeds in 1-year timesteps for the remainder of the agent’s lifetime. This study used deidentified data from publicly available databases; thus, institutional review board approval and informed consent was not required per the City University of New York. We conducted this study from April 5, 2024, to September 10, 2024. The study follows the Consolidated Health Economic Evaluation Reporting Standards (CHEERS) reporting guidelines.

### Agent Physical Activity

Every week, each agent draws a mean number of days per week that they are physically active for 60 minutes or more from an age- and sex-specific distribution calculated as a weighted mean (Table 1)^23,26,27,28,29,30,31^ based on National Survey of Children’s Health data (2016-2020 [5 years combined]). We assume agents are physically active for less than 60 minutes on all other days (ie, non-PA days). This PA translates into additional daily caloric expenditure, used in the agent’s metabolic model to determine weight gain and loss until 18 years of age (eFigure in Supplement 1).

**Table 1.** Table of Inputs

Input parameter	Value	Source
**Population size (N = 8 299 353), No.**			
Ages 6-11 y		
Male	2 078 664	US Census,^26^ 2022
Female	1 992 402	
Ages 12-17 y		
Male	2 161 455	US Census,^26^ 2022
Female	2 066 832	
**Physical activity level, mean (SD)**			
Days per wk with ≥60 min of physical activity, No.^a^		
Ages 6-11 y		
Male	4.16 (0.08)	National Survey of Children’s Health^27^ (2016-2020)
Female	3.97 (0.07)	
Ages 12-17 y		
Male	3.68 (0.08)	National Survey of Children’s Health^27^ (2016-2020)
Female	3.07 (0.07)	
Physical activity intensity, metabolic equivalents		
Male	5.3 (1.6)	Harrell et al,^28^ 2003
Female	4.3 (1.56)	
**Sports participation**			
Sports participation, % youth who participate on a regular basis		
Male	40.2	Project Play,^29^ 2023
Female	34.5	
Moderate-vigorous physical activity, mean min/wk^b^		
Male	418.49	Kanters et al^30^; Sports and Fitness Industry Association, ^31^ 2024
Female	320.59	
Metabolic equivalents, mean (range)		
Male	3.95 (3.02-4.84)	Kanters et al^30^
Female	3.55 (2.41-4.35)	
**Metabolic parameters, mean (SD)**			
Fat free mass, kg		
Ages 6-11 y		
Male^c^	26.12 (6.45)	NHANES^23^ (2017-2020)
Female^c^	32.42 (10.82)	
Ages 12-17 y		
Male^c^	52.94 (11.41)	NHANES^23^ (2017-2020)
Female^c^	40.92 (6.73)	
Fat tissue and fat mass ratio		
Ages 6-11 y		
Male^c^	7.35 (3.66)	NHANES^23^ (2017-2020)
Female^c^	15.20 (7.84)	
Ages 12-17 y		
Male^c^	13.11 (5.18)	NHANES^23^ (2017-2020)
Female^c^	20.21 (6.90)	
**Overweight and obesity prevalence, mean (95% CI)**		
Overweight prevalence		
Ages 6-11 y		
Male	12.79 (9.83-15.74)	NHANES,^23^ 2017-2020
Female	13.58 (10.35-16.80)	
Ages 12-17 y		
Male	14.45 (10.86-18.03)	NHANES,^23^ 2017-2020
Female	18.21 (14.39-22.03)	
Obesity prevalence			
Ages 6-11 y		
Male	23.72 (19.79-27.66)	NHANES,^23^ 2017-2020
Female	17.58 (13.72-20.98)	
Ages 12-17 y		
Male	21.77 (17.95-25.58)	NHANES,^23^ 2017-2020
Female	20.23 (16.54-23.91)	

Abbreviation: NHANES, National Health and Nutrition Examination Survey.

^a^ Mean number of days was calculated from the question, “During the past week, on how many days did this child exercise, play a sport, or participate in physical activity for at least 60 minutes?” which included response options of 0 days, 1 to 3 days, 4 to 6 days, and every day (7 days). To determine mean physical activity days per week, we calculated the weighted mean by multiplying the proportion of children for each response option by the number of days they performed at least 60 minutes of PA and then summed them. For response options with a range of days, we used the mean of the range (eg, 1-3 days was estimated at 2 days, 4-6 days was estimated at 5 days). This was done for each sex and age group.

^b^ Calculated using observational studies and surveys. We used reported minutes spent physically active as well as the intensity level (measured metabolic equivalents) of physical activity from observed youth sports practices and games. We then calculated the mean distribution of minutes of physical activity weighted by the approximate proportion participating in each sport by using the metabolic equivalents and the reported proportion of male and female youth participating in each of these sports.

^c^ Values are mean (SD) across population; each agent has its own value.

### Agent Sports Participation

Each agent has a sex-specific probability of sports participation (Table 1). Individuals participating in sports engage in sex-specific moderate-vigorous physical activity minutes per week (Table 1) and in nonsport PA (assumed to be the same regardless of participation). Again, this affects daily caloric expenditures and weight gains and losses (eFigure in Supplement 1).

### Agent Physical Health Outcomes

An embedded Markov model (eFigure, eMethods, and eTables 1-3 in Supplement 1), described in previous publications,^13,18,19,32^ determines the physical health outcomes and weight-related health conditions (eg, stroke, coronary heart disease, type 2 diabetes and its complications [neuropathy, retinopathy, and nephropathy], and cancers) and death from these outcomes that each agent experiences over time.

### Economic Outcomes

Agents accrue relevant costs and health effects (measured in quality-adjusted life-years [QALYs]) as they proceed through the model. We report costs from the third-party payer perspective, which includes direct medical costs, as well as the societal perspective, which includes both direct and indirect (ie, productivity losses due to presenteeism) costs. Daily wages across all occupations^33^ serve as a proxy for productivity losses, with presenteeism estimated as daily wages multiplied by the individual’s health condition–specific utility weight (eTable 1 in Supplement 1) for the duration of their condition (assumed to be the remainder of their lifetime) as follows: daily wage × (1 − utility weight) × outcome duration. The utility weight serves as a proxy for the reduction in productivity due to the agent’s specific health condition (eg, a utility weight of 0.6 means an agent is 40% less productive than they would have been if perfectly healthy). Every agent accrues productivity losses because everyone contributes to society, regardless of age or employment status. All costs are reported in 2024 US dollars. Because cost data come from various sources published in different years, we converted all past and future costs using a standard 3% rate, regardless of year, following recommendations from the Panel on Cost-Effectiveness in Health and Medicine.^34,35^ All future QALYs are presented in net present value, discounted with a 3% rate.

### Experimental Scenarios, Sensitivity Analysis, and Statistical Analysis

Our first set of simulation experiments explored what would happen if PA disparities between male and female youths were eliminated within each age group (eg, all female youths aged 12-17 increase PA to 3.68 days per week of ≥60 minutes of PA, meeting that of male youths in the same age group). Different scenarios explored what would happen if sex disparities were reduced by varying degrees (25%-75%). Sensitivity analyses explored what would happen if female youths had the same PA minutes and PA intensity (eg, metabolic equivalents) as their male counterparts. Lastly, we varied the amount of PA agents received on non-PA days (0-45 minutes). Another set of experiments explored what would happen if youth sports participation disparities were eliminated between male and female youths (eg, bringing female sports participation up to the level of male sports participation). Different scenarios explored reducing the sex disparities by varying degrees (25%-75%). Statistical analyses, including 95% CI calculations, were performed using Python, version 3.7 (Python Software Foundation).

## Results

This economic evaluation modeled 8 299 353 US children (aged 6-11 years; n = 4 071 066) and adolescents (aged 12-17 years; n = 4 228 287), including 4 240 119 (51.1%) male and 4 059 234 (48.9%) female participants. Physical health outcomes and economic outcomes for youth PA and sports participation are reported.

### Physical Health Outcomes When Reducing Sex Disparities in Youth PA Levels

**Figure 1.**
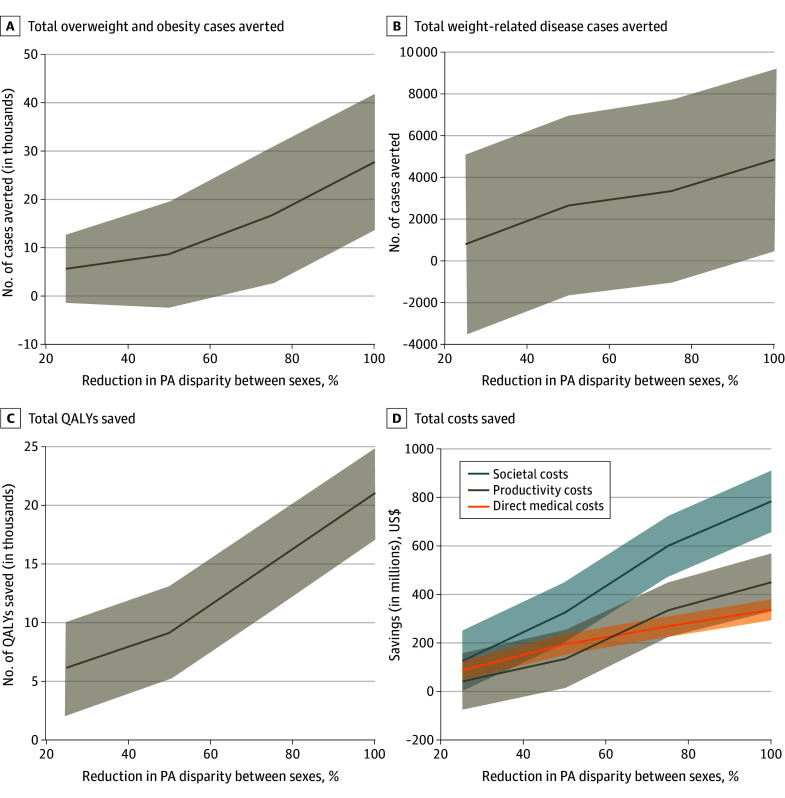
Economic and Clinical Outcomes of Reducing Physical Activity (PA) Sex Disparities in US Youth Lines indicate means; shaded bands, 95% CIs. QALY indicates quality-adjusted life-year.

Table 2 gives the physical health outcomes (averted overweight and obesity cases and weight-related disease cases) resulting from reducing or eliminating the sex disparities in PA levels among youth. Bringing all US female children and adolescents up to the observed PA levels of their male counterparts resulted in an absolute decrease in overweight and obesity prevalence in all children and adolescents of 0.338% (95% CI, 0.337%-0.345%), with 28 061 (95% CI, 25 358-30 763) fewer overweight and obesity cases in female participants (Figure 1). This averted 4869 (95% CI, 4007-5732) weight-related disease cases during their lifetimes.

**Table 2.** Clinical Outcomes in Female Children and Adolescents When Reducing Existing PA Sex Disparities Compared With Current Levels of PA

Outcome	Mean (95% CI), No.^a^			
	25% Reduction	50% Reduction	75% Reduction	100% Reduction
**Health effects**				
Female QALYs saved				
Ages 6-11 y	3115 (295 to 5936)	2669 (−60 to 5399)	6706 (3769 to 9642)	6822 (3900 to 9744)
Ages 12-17 y	3221 (358 to 6083)	6226 (3163 to 9290)	8128 (4962 to 11 295)	14 013 (10 914 to 17 113)
Total	6336 (2318 to 10 355)	8896 (4793 to 12 999)	14 834 (10 516 to 19 153)	20 835 (16 576 to 25 095)
Female years of life saved				
Ages 6-11 y	8306 (−7467 to 24 078)	−5383 (−20 048 to 9282)	−8929 (−24 357 to 6499)	10 034 (−3945 to 24 013)
Ages 12-17 y	5944 (−8340 to 20 229)	−929 (−14 941 to 13 083)	3719 (−11 858 to 19 295)	3467 (−10 982 to 17 917)
Total	14 250 (−7030 to 35 530)	−6313 (−26 595 to 13 970)	−5210 (−27 134 to 16 713)	13 501 (−6604 to 33 606)
**Medical conditions**				
Female overweight and obesity cases averted				
Ages 6-11 y	1826 (908 to 2745)	2723 (1248 to 4198)	3022 (1100 to 4943)	8667 (7014 to 10 320)
Ages 12-17 y	4099 (3191 to 5008)	6304 (4830 to 7777)	13 572 (11 634 to 15 510)	19 394 (17 256 to 21 532)
Total	5926 (4634 to 7217)	9027 (6942 to 11 112)	16 594 (13 865 to 19 323)	28 061 (25 358 to 30 763)
**Total cases of weight-related diseases (cancer, CHD, diabetes, and stroke) averted**				
Female cancer cases averted				
Ages 6-11 y	−21 (−376 to 334)	274 (−66 to 613)	325 (10 to 640)	430 (118 to 743)
Ages 12-17 y	140 (−182 to 463)	226 (−118 to 570)	518 (192 to 843)	844 (474 to 1213)
Total	119 (−360 to 599)	500 (16 to 983)	843 (390 to 1296)	1274 (790 to 1758)
Female CHD cases averted				
Ages 6-11 y	299 (−66 to 665)	296 (−46 to 638)	348 (−5 to 701)	531 (174 to 887)
Ages 12-17 y	168 (−163 to 499)	536 (166 to 906)	799 (406 to 1192)	834 (467 to 1201)
Total	467 (−26 to 960)	832 (328 to 1336)	1147 (618 to 1675)	1365 (853 to 1876)
Female diabetes cases averted				
Ages 6-11 y	−83 (−420 to 253)	953 (625 to 1280)	493 (156 to 831)	876 (528 to 1224)
Ages 12-17 y	301 (−50 to 651)	378 (32 to 725)	873 (492 to 1253)	1355 (998 to 1711)
Total	218 (−269 to 704)	1331 (855 to 1807)	1366 (857 to 1875)	2231 (1732 to 2729)
Female stroke cases averted				
Ages 6-11 y	−62 (−240 to 117)	−136 (−316 to 43)	−68 (−237 to 101)	−38 (−232 to 155)
Ages 12-17 y	25 (−161 to 211)	−37 (−231 to 156)	173 (−21 to 367)	−72 (−266 to 122)
Total	−37 (−295 to 221)	−174 (−438 to 90)	105 (−153 to 362)	−110 (−384 to 163)
**Total deaths from weight-related diseases (cancer, CHD, diabetes, and stroke) averted**				
Female cancer deaths averted				
Ages 6-11 y	−32 (−360 to 295)	283 (−30 to 596)	384 (71 to 697)	348 (25 to 670)
Ages 12-17 y	164 (−184 to 513)	318 (−31 to 667)	428 (73 to 782)	1003 (673 to 1334)
Total	132 (−347 to 611)	601 (133 to 1070)	812 (339 to 1285)	1351 (889 to 1813)
Female CHD deaths averted				
Ages 6-11 y	51 (−117 to 220)	167 (4 to 330)	357 (182 to 532)	257 (85 to 428)
Ages 12-17 y	−24 (−198 to 149)	194 (4 to 383)	247 (52 to 441)	456 (292 to 620)
Total	27 (−215 to 269)	361 (111 to 611)	604 (342 to 866)	713 (475 to 951)
Female diabetes deaths averted				
Ages 6-11 y	26 (−26 to 77)	36 (−12 to 84)	50 (5 to 96)	43 (−2 to 87)
Ages 12-17 y	11 (−39 to 61)	−6 (−53 to 42)	9 (−40 to 59)	57 (8 to 106)
Total	37 (−35 to 109)	30 (−37 to 97)	60 (−7 to 126)	99 (33 to 166)
Female stroke deaths averted				
Ages 6-11 y	10 (−98 to 118)	40 (−65 to 145)	38 (−70 to 147)	14 (−100 to 128)
Ages 12-17 y	25 (−75 to 125)	−8 (−130 to 115)	−3 (−111 to 105)	−34 (−145 to 76)
Total	35 (−112 to 182)	32 (−129 to 194)	35 (−118 to 188)	−20 (−179 to 138)

Abbreviations: CHD, coronary heart disease; PA, physical activity; QALYs, quality-adjusted life-years.

^a^ The 95% CIs that include zero are not significant.

Because it is unlikely for the PA disparity between male and female participants to disappear entirely, experiments simulated reducing the disparity by different degrees (25%-75%) (Table 2). For example, when reducing PA sex disparities by 50%, overweight and obesity prevalence decreased by an absolute 0.109% (95% CI, 0.104%-0.114%), with 9027 (95% CI, 6942-11 112) fewer overweight and obesity cases in female participants and 2663 (95% CI, 1817-3508) fewer weight-related disease cases during their lifetimes.

The benefits in physical health outcomes from eliminating PA disparities are not evenly distributed among age groups. Compared with other age groups, 12- to 17-year-olds experienced the greatest benefits (per 10 000 persons) when completely eliminating the disparity, averting 50.3 (95% CI, 37.1-63.6) more overweight and obesity cases and 5.4 (95% CI, 1.2-9.7) more weight-related disease cases and gaining 33.6 (95% CI, 29.5-37.7) more QALYs per 10 000 persons. This is because female youths aged 12 to 17 years started with the lowest PA levels compared with male youths in the same age category.

Given that in some situations not only are male children and adolescents physically active for more time than female children and adolescents, but they also exercise at a higher intensity, we explored what would happen when eliminating disparities in both the amount of time physically active and PA intensity level. Eliminating both disparities resulted in a decrease in overweight and obesity prevalence of 1.18% (95% CI, 1.17%-1.20%), with 98 186 (95% CI, 93 338-103 034) fewer overweight and obesity cases across the 6- to 17-year-old population. This averted 16 371 (95% CI, 15 513-17 228) weight-related disease cases during their lifetimes.

If individuals were less active on non-PA days (ie, physically active <60 minutes), the disparity between male and female children and adolescents became larger, thus increasing the impact of eliminating the disparity. For example, when female children and adolescents got 0 PA minutes on non-PA days (vs 30 minutes), eliminating sex disparities decreased overweight and obesity prevalence by an absolute 0.288% (95% CI, 0.279%-0.297%), with 23 929 (95% CI, 21 092-26 765) fewer overweight and obesity cases and 4530 (95% CI, 3676-5384) fewer weight-related diseases cases. Conversely, when getting 45 PA minutes on non-PA days, eliminating disparities decreased overweight and obesity prevalence by 0.049% (95% CI, 0.041%-0.057%), with 4072 (95% CI, 1571-6572) fewer overweight and obesity and 1552 (95% CI, 730-2374) fewer weight-related disease cases.

### Economic Outcomes When Reducing Sex Disparities in Youth PA Levels

Figure 1D shows the recurring cost savings for every new cohort of 6- to 17-year-olds resulting from the improved physical health outcomes achieved by eliminating sex disparities in PA. Completely eliminating disparities saved $779.87 million (95% CI, $653.23 million to $906.51 million) in societal costs, of which 43% was direct medical cost-savings and 57% was productivity losses averted.

As with physical health outcomes, cost savings are not equally distributed, with 12- to 17-year-olds garnering the greatest cost savings. When eliminating disparities (eg, 100% reduction), the 12- to 17-year-old age group saved $219.96 million (95% CI, $188.37 million to $251.55 million) in direct medical costs and $280.66 million (95% CI, $193.01 million to $368.31 million) in productivity losses during their lifetimes, approximately twice as much as the 6- to 11-year-old group. Although the least amount of savings occurred in the 6- to 11-year-old group, increasing PA to 4.16 days per week of 60 minutes of PA per day (the levels that male youths in that age group currently get) for everyone within that age group still saved $113.49 million (95% CI, $83.98 million to $143.01 million) in direct medical costs and $165.76 million (95% CI, $85.23 million to $246.29 million) in productivity losses.

Cost savings increased approximately linearly (Figure 1D), with the greater disparity reductions leading to higher amounts of savings. For example, reducing PA disparity by 25% saved $83.80 million (95% CI, $44.24 million to $123.37 million) in direct medical costs and $38.45 million (95% CI, −$77.88 million to $154.78 million) in productivity losses, and increasing this to a 50% reduction saved $107.79 million and $92.83 million more in direct medical costs and productivity losses, respectively. Increasing the disparity reduction from 75% to 100%, saved an additional $67.45 million and $114.96 million in direct medical costs and productivity losses, respectively.

Reducing PA disparities in both days active per week and PA intensity between male and female youth saved $2.47 billion (95% CI, $2.35 billion to $2.60 billion), of which $1.18 billion (95% CI, $1.13 billion to $1.23 billion) were direct medical costs and $1.29 billion (95% CI, $1.41 billion to $1.78 billion) were productivity losses. Similarly, eliminating the disparity when female youth had 0 minutes PA on non-PA days saved $707.01 million (95% CI, $577.37 million to $836.65 million), of which $319.30 million (95% CI, $275.72 million to $362.88 million) were direct medical costs and $387.71 million (95% CI, $265.61 million to $509.8 million) were productivity losses. Conversely, when female children and adolescents had 45 PA minutes on non-PA days, eliminating the disparity saved $132.83 million (95% CI, $11.17 million to $254.49 million) in societal costs, of which $72.21 million (95% CI, $31.66 million to $112.76 million) were direct medical costs and $60.62 million (95% CI, –$54.08 million to $175.32 million) were productivity losses. Thus, similar to health outcomes, the disparity is smaller if individuals are more active on non-PA days.

### Physical Health Outcomes When Reducing Sex Disparities in Youth Sports Participation

When only closing the sports participation gap among male and female children and adolescents in the US, bringing females’ sports participation level (35.4%) up to the same level as that of males (40.2%) resulted in an absolute decrease in overweight and obesity prevalence in all children and adolescents of 1.02% (95% CI, 0.93%-1.22%), with 41 499 (95% CI, 37 874-45 125) fewer overweight and obesity cases in 6- to 17-year-old female participants. Eliminating the sports participation disparity averted 8939 (95% CI, 8088-9790) cases of weight-related diseases over the course of their lifetimes (Table 3).

**Table 3.** Clinical Outcomes in Female Children and Adolescents When Reducing Existing Sports Participation Disparities Compared With Current Levels of PA

Outcome	Mean (95% CI), No.^a^			
	25% Reduction	50% Reduction	75% Reduction	100% Reduction
**Health effects**				
Female QALYs saved				
Ages 6-11 y	6247 (3473 to 9021)	11 341 (8626 to 14 055)	12 643 (9968 to 15 319)	18 112 (15 035 to 21 189)
Ages 12-17 y	3964 (1094 to 6834)	12 558 (9730 to 15 387)	13 009 (10 022 to 15 995)	22 658 (19 739 to 25 577)
Total	10 211 (6220 to 14 202)	23 899 (19 979 to 27 819)	25 652 (21 643 to 29 661)	40 770 (36 528 to 45 012)
Female years of life saved				
Ages 6-11 y	−3702 (−18 012 to 10 608)	3442 (−10 674 to 17 558)	−3692 (−18 180 to 10 796)	5388 (−7985 to 18 760)
Ages 12-17 y	−4770 (−18 628 to 9088)	14 270 (157 to 28 383)	9510 (−6060 to 25 080)	6254 (−7858 to 20 367)
Total	−8472 (−28 393 to 11 449)	17 712 (−2249 to 37 673)	5818 (−15 450 to 27 086)	11 642 (−7800 to 31 084)
**Medical conditions**				
Female overweight and obesity cases averted				
Ages 6-11 y	3321 (2177 to 4464)	9364 (7654 to 11 075)	12 851 (10 796 to 14 906)	18 695 (16 216 to 21 175)
Ages 12-17 y	3548 (2362 to 4734)	11 058 (9124 to 12 991)	14 330 (11 915 to 16 745)	22 804 (20 160 to 25 448)
Total	6869 (5221 to 8516)	20 422 (17 840 to 23 003)	27 181 (24 010 to 30 352)	41 499 (37 874 to 45 125)
**Total cases of weight-related diseases (cancer, CHD, diabetes, and stroke) averted**				
Female cancer cases averted				
Ages 6-11 y	124 (−211 to 459)	312 (4 to 621)	814 (461 to 1167)	601 (266 to 937)
Ages 12-17 y	51 (−308 to 410)	477 (104 to 849)	874 (504 to 1244)	1187 (834 to 1540)
Total	175 (−316 to 666)	789 (305 to 1273)	1688 (1177 to 2199)	1788 (1301 to 2275)
Female CHD cases averted				
Ages 6-11 y	−93 (−462 to 276)	882 (535 to 1229)	954 (579 to 1330)	1342 (1008 to 1676)
Ages 12-17 y	483 (132 to 833)	888 (513 to 1262)	991 (603 to 1379)	1618 (1250 to 1986)
Total	389 (−120 to 898)	1770 (1259 to 2281)	1946 (1405 to 2486)	2960 (2463 to 3456)
Female diabetes cases averted				
Ages 6-11 y	−16 (−331 to 298)	1219 (875 to 1563)	1111 (776 to 1445)	1872 (1530 to 2214)
Ages 12-17 y	264 (−116 to 644)	844 (504 to 1184)	1555 (1172 to 1937)	2319 (1969 to 2670)
Total	247 (−246 to 741)	2063 (1579 to 2547)	2665 (2157 to 3173)	4191 (3701 to 4681)
Female stroke cases averted				
Ages 6-11 y	134 (−46 to 313)	21 (−159 to 202)	17 (−175 to 209)	−65 (−244 to 114)
Ages 12-17 y	−87 (−256 to 81)	0 (−198 to 197)	−117 (−308 to 73)	−25 (−214 to 164)
Total	46 (−200 to 293)	21 (−247 to 289)	−100 (−371 to 170)	−90 (−350 to 170)
**Total deaths of weight-related diseases (cancer, CHD, diabetes, and stroke) averted**				
Female cancer deaths averted				
Ages 6-11 y	163 (−162 to 489)	634 (357 to 911)	651 (347 to 956)	812 (499 to 1125)
Ages 12-17 y	213 (−107 to 534)	835 (509 to 1161)	739 (429 to 1049)	1341 (1022 to 1660)
Total	377 (−80 to 833)	1468 (1041 to 1896)	1390 (956 to 1825)	2153 (1706 to 2600)
Female CHD deaths averted				
Ages 6-11 y	196 (29 to 364)	266 (96 to 437)	524 (351 to 697)	507 (334 to 679)
Ages 12-17 y	178 (−4 to 360)	312 (129 to 494)	459 (269 to 649)	797 (622 to 971)
Total	374 (127 to 621)	578 (328 to 828)	983 (726 to 1239)	1303 (1058 to 1548)
Female diabetes deaths averted				
Ages 6-11 y	7 (−41 to 55)	8 (−40 to 56)	37 (−10 to 84)	68 (22 to 115)
Ages 12-17 y	34 (−19 to 86)	14 (−39 to 66)	60 (11 to 109)	128 (76 to 180)
Total	40 (−30 to 111)	21 (−49 to 92)	97 (29 to 165)	196 (126 to 266)
Female stroke deaths averted				
Ages 6-11 y	−20 (−126 to 87)	−94 (−200 to 13)	−3 (−116 to 111)	−17 (−121 to 88)
Ages 12-17 y	41 (−67 to 149)	−1 (−111 to 109)	−46 (−166 to 75)	18 (−94 to 130)
Total	22 (−130 to 173)	−95 (−247 to 58)	−49 (−214 to 117)	1 (−152 to 154)

Abbreviations: CHD, coronary heart disease; PA, physical activity; QALYs, quality-adjusted life-years.

^a^ The 95% CIs that include zero are not significant.

Table 3 also indicates what happens when reducing the sports participation disparity to varying degrees. For example, when reducing the disparities by 50%, overweight and obesity prevalence in the entire 6- to 17-year-old population decreased by an absolute 0.50% (95% CI, 0.44%-0.60%), with 20 422 (95% CI, 17 840-23 003) fewer cases of overweight and obesity in female 6- to 17-year-olds and averted 4622 (95% CI, 3768-5476) cases of weight-related diseases over their lifetimes.

### Economic Outcomes When Reducing Sex Disparities in Youth Sports Participation

**Figure 2.**
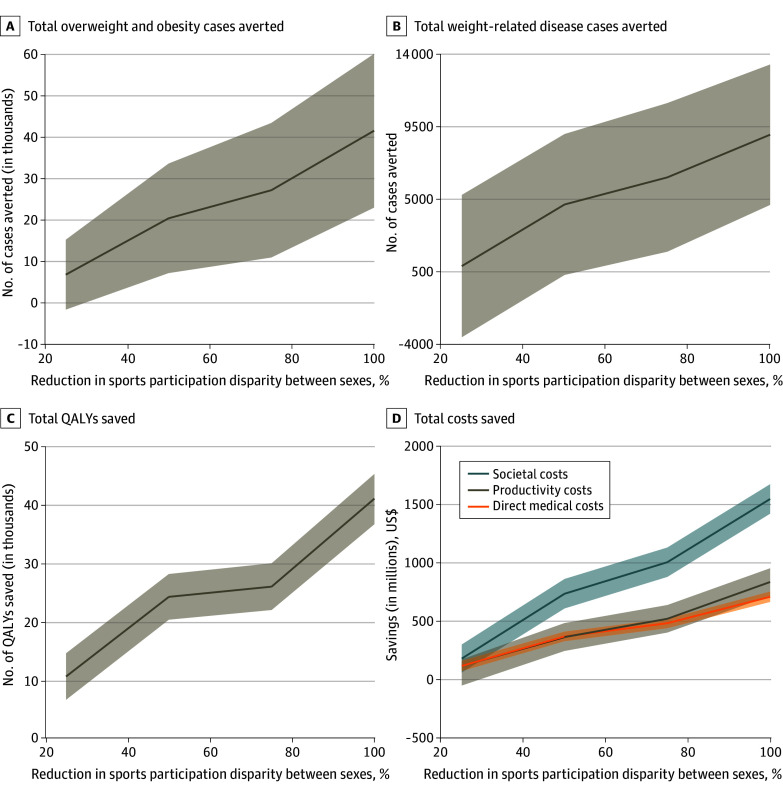
Economic and Clinical Outcomes of Reducing Sports Participation Sex Disparities in US Youth Lines indicate means; shaded bands, 95% CIs. QALY indicates quality-adjusted life-year.

The improved physical health outcomes achieved by eliminating the disparity in sports participation between male and female youth saved $1.55 billion (95% CI, $1.43 billion to $1.68 billion) in societal cost savings (46% from direct medical cost savings and 54% from productivity losses averted), which recur for every new cohort of 6- to 17-year-olds (Figure 2). Similar to eliminating PA disparities, the cost savings increased relatively linearly, with greater reductions in the sports participation disparity (Figure 2). For example, reducing the sports participation disparity by 25% saved $118.81 million (95% CI, $75.09 million to $162.53 million) in direct medical costs and $63.40 million (95% CI, –$49.06 million to $175.86 million) in productivity losses averted, and increasing this to a 50% reduction resulted in $253.21 million and $304.01 million more savings in direct medical costs and productivity losses, respectively. Increasing the sports participation disparity reduction from 75% to 100% resulted in an additional $227.16 million and $315.91 million in savings in direct medical costs and productivity losses, respectively.

## Discussion

This simulation study found that reducing or eliminating existing sex disparities could save $122 million to $780 million for PA and even more ($182 million to $1550 million) for sports participation. Such cost savings would recur for every new cohort of 6- to 17-year-olds. To put these cost savings in perspective, they significantly exceed the costs of many existing efforts to increase PA, such as the $4.5 million spent from 2019 to 2020 on Youth Engagement in Sports Initiative grants that aim to expand youth participation in sports and encourage regular PA^36^ and the $1 billion spent from 2015 to 2022 on the Safe Routes to School programs that support safe walking and biking to school.^37^

Having these numbers can help pave the way for more investment into reducing sex disparities in PA, and studies have identified paths toward decreasing such disparities by identifying their root causes. One identified root cause is gender norms and expectations, which define girlhood and what is expected from girls (eg, supposed to be less active than boys, more aware of how others perceive them).^38,39^ One way to address this root cause is to increase the number of female coaches and trainers, because they can serve as important role models and can enhance girls’ social inclusion and help overcome cultural barriers.^40^ Additionally, spotlighting women athletes as role models can also help increase sports participation by increasing interest, promoting PA, and shifting norms.^41^ Another root cause is psychosocial behaviors (eg, need for support from friends and parents,^42^ fear of judgment^38,43^). Providing educators and parents with information and tools on engaging girls in PA and sports can help increase support. Mentoring programs (eg, pairing girls with female student athletes^44^) can also provide support to younger girls and reduce anxieties about judgment when engaging in PA. Another root cause is inequities in investments (eg, booster clubs,^45^ scholarship funding^46,47,48^). Investing in underresourced schools or communities may help create a more engaging environment for PA.^49^ Investments into sports budgets can provide more opportunities to play, and offering more scholarships can provide motivation to continue to play.

Of the different ways to reduce disparities in PA, reducing those in sports may be the easiest and most effective to implement. Sports participation may be easier to measure and monitor (eg, attendance at games and practices, sports team rosters, timing moderate-vigorous physical activity minutes) than overall PA. Plus, sports-related policies and interventions (eg, investing in after-school sports programming, revitalizing local neighborhood sports leagues) may be more readily implemented because they have clear implementation locations and settings (eg, schools, athletic associations, gyms), specific organizing bodies, existing demand among the general public, and many funding pathways that do not require the use of school budgets. Additionally, sports participation has added benefits beyond increased PA, such as helping to reduce stress and anxiety, increasing self-esteem and confidence, and helping children make friends and build social connections.^50,51,52^

### Limitations

This study has some limitations. As a simplification of real life, a model cannot account for every possible event or outcome. To remain conservative about the impact of increasing PA, we did not include other potential physical health (eg, improved bone health) and mental health (eg, decreases in depression and anxiety) benefits.^53,54,55,56,57,58^ Additionally, we did not represent underweight as an adult body mass index category because its prevalence is only 3.4% among 18- to 19-year-olds^59^ and decreases with age,^60^ although those in this category can benefit from increased PA (eg, stimulate appetite; build strong bones, muscles, and joints^61,62,63,64^). Furthermore, the model divided the youth population by biological sex (due to data availability) and did not distinguish those who are nonbinary or transgender and could benefit from greater inclusiveness. Our model also did not explicitly represent how differences between female and male PA and sports participation levels may vary by other sociodemographic characteristics (eg, race, ethnicity, and/or socioeconomic status). However, because the goal was to generate national averages and totals using data from representative samples, these samples should have accounted for these variations. Future studies could explore the benefits of reducing PA and sports disparities for nonbinary youth^65^ and among sexes and genders of different races, ethnicities, and socioeconomic statuses.

## Conclusions

This simulation study found that reducing or eliminating existing sex disparities in youth PA and sports participation could avert 28 061 to 41 499 overweight and obesity cases by 18 years of age as well as 4869 to 8939 weight-related disease cases in youths’ lifetimes and save $780 million to $1550 million for each new cohort of 6- to 17-year-olds. The cost savings could substantially exceed the cost of programs that target greater equity.

## Notes

Supplement 1. **eMethods.** Agent Physical Health Outcomes **eFigure.** Youth Physical Activity Disparity Model **eTable 1.** Embedded Markov Model Table of Inputs **eTable 2.** Transition Probabilities for Adults Age ≤45 Years Old **eTable 3.** Transition Probabilities for Adults Age >45 Years Old
eReferences.

Supplement 2.
Data Sharing Statement
