# Supplementary material for: Modeling Health and Economic Outcomes of Eliminating Sex Disparities in Youth Physical Activity
Source: JAMA Netw Open. 2024 Nov 25;7(11):e2446775. doi: 10.1001/jamanetworkopen.2024.46775 (PMC11589798; doi:10.1001/jamanetworkopen.2024.46775)
Supplement: Supplement 1. — eMethods. Agent Physical Health Outcomes eFigure. Youth Physical Activity Disparity Model eTable 1. Embedded Markov Model Table of Inputs eTable 2. Transition Probabilities for Adults Age ≤45 Years Old eTable 3. Transition Probabilities for Adults Age >45 Years Old eReferences. [file jamanetwopen-e2446775-s001.pdf]

Supplementary Online Content

Tamura K, Martinez MF, Deng Y, et al. Modeling health and economic outcomes of eliminating sex disparities in youth physical activity. *JAMA Netw Open*. 2024;7(11):e2446775. doi:10.1001/jamanetworkopen.2024.46775

eMethods. Agent Physical Health Outcomes
eFigure. Youth Physical Activity Disparity Model
eTable 1. Embedded Markov Model Table of Inputs
eTable 2. Transition Probabilities for Adults Age ≤45 Years Old
eTable 3. Transition Probabilities for Adults Age >45 Years Old
eReferences.

This supplementary material has been provided by the authors to give readers additional information about their work.

© 2024 Tamura K et al. *JAMA Network Open.*

eMethods.

Agent Physical Health Outcomes

An embedded Markov model (eFigure), described in previous publications,[1-4] determines the physical health outcomes that each agent experiences over time. It consists of 15 mutually exclusive health states which account for anthropometric measures (e.g., BMI) and the presence and severity of risk factors associated with weight. At age 18, agents start at a metabolically healthy state and one of 3 states based on their BMIs [normal weight (18.5≤BMI<25), overweight (25≤BMI<30), or obese (BMI≥30)]. Each simulated year, the agent has probabilities of staying in the same state, moving to a new health state, or dying based on their current health state-, age-, and sex-specific probabilities (Supplement). In each year, the agent has health state-specific probabilities of developing weight-related health outcomes such as stroke, coronary heart disease (CHD), type 2 diabetes (and its complications: neuropathy, retinopathy, nephropathy), and cancers as well as probabilities of dying from each health outcome (eTable 1). Individuals accrue age-specific medical costs (eTable 1) based on their health state (e.g., chronic health state 1, which include routine medical care/office visits) and their health outcome (e.g., costs due to medications, treatments, testing, office visits, hospitalization) as well as lost productivity and quality-adjusted life-years (QALYs).

© 2024 Tamura K et al. *JAMA Network Open.*

eFigure. Youth Physical Activity Disparity Model

Notes: BMI indicates body mass index (calculated as weight in kilograms divided by height in meters squared); CHS, chronic health state; MVPA, moderate to vigorous physical activity; QALYs, quality-adjusted life-years.

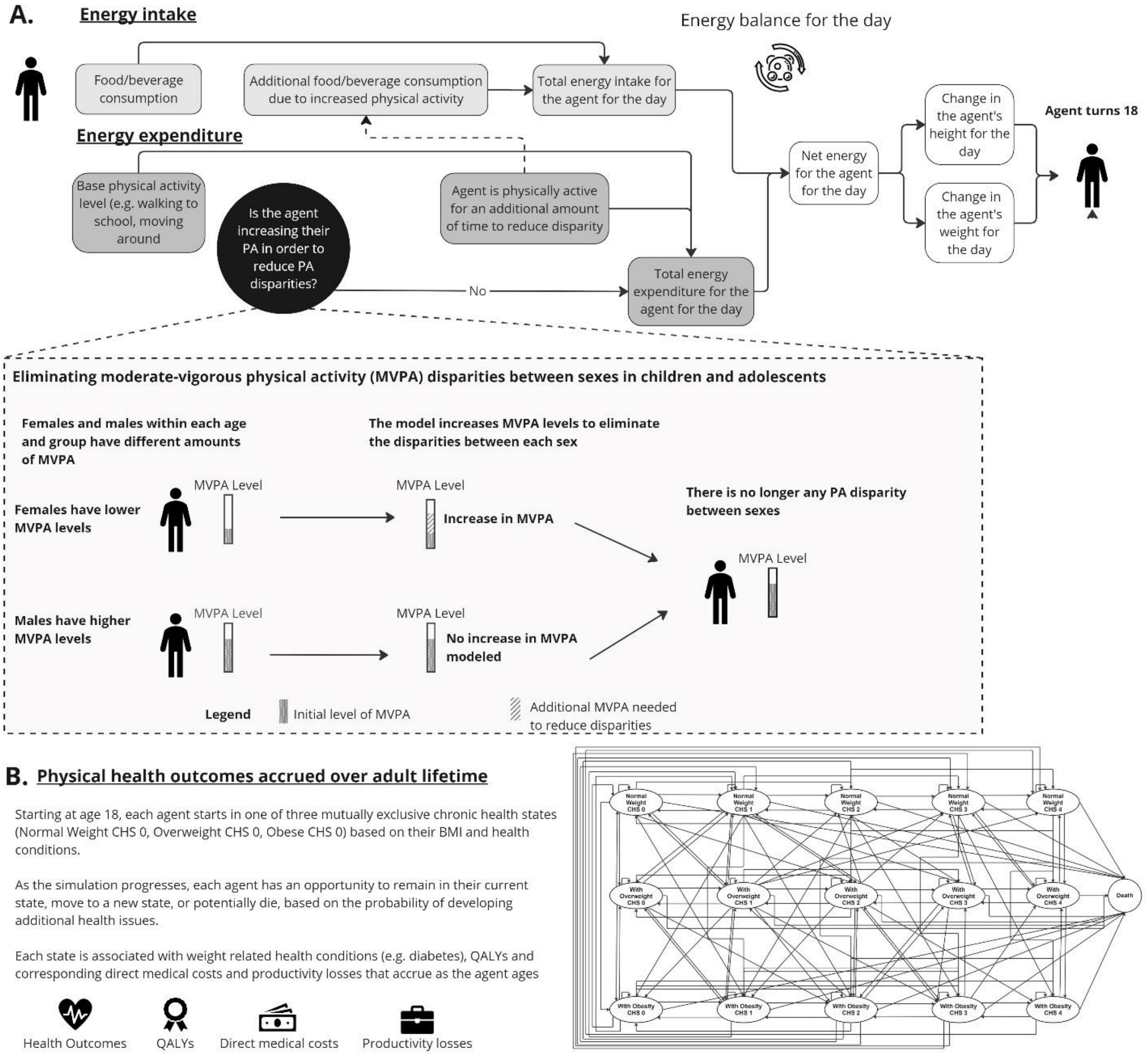

© 2024 Tamura K et al. *JAMA Network Open.*

eTable 1. CEOM Table of Inputs

Cost Values (2024 Dollars)	Distribution type	Mean	Range or standard deviation	Source
Chronic Health State 1	Point Estimate	$629.59		5
Chronic Health State 2	Point Estimate	$1,422.58		5
Normal Weight Chronic Health State 3	Point Estimate	$2,128.18		5
Overweight Chronic Health State 3	Point Estimate	$2,603.21		5
Obese Chronic Health State 3	Point Estimate	$4,978.40		5
Normal Weight Chronic Health State 4	Point Estimate	$3,681.23		5
Overweight Chronic Health State 4	Point Estimate	$4,631.31		5
Obese Chronic Health State 4	Point Estimate	$9,381.70		5
Heart Disease: Age 18-44	Gamma	$6,034.99	$2,775.73	6
Heart Disease: Age 45- 65	Gamma	$8,124.99	$1,368.92	6
Heart Disease: Age 65+	Gamma	$5,556.27	$448.21	6
Diabetes Mellitus: Age 18-44	Gamma	$5,965.17	$1,232.79	6
Diabetes Mellitus: Age 44-65	Gamma	$5,844.57	$401.07	6
Diabetes Mellitus: Age 65+	Gamma	$4,660.48	$236.70	6
Hypertension: Age 18-44	Gamma	$834.90	$326.17	6
Hypertension: Age 44-65	Gamma	$688.69	$79.52	6
Hypertension: Age 65+	Gamma	$859.42	$89.67	6
Diabetic Nephropathy	Triangular	$1,157.21	$1,041 - $1,273	7
End-Stage Renal Disease (ESRD)-Initial year	Triangular	$89,409.29	$80,469 - $98,351	8
Diabetic Neuropathy	Triangular	$16,299.22	$14,669 - $17,929	9
Diabetic Retinopathy	Triangular	$5,038.62	$4,535 - $5,542	10
Blindness	Triangular	$9,246.68	$8,322 - $10,171	10
Cerebrovascular Disease (CVD), including Stroke: Age 18-64	Gamma	$11,519.22	$4,456.69	6
CVD, including Stroke: Age 65+	Gamma	$8,362.97	$1,415.47	6
Breast Cancer: First Year, Females	Point Estimate	$40,658.67		11
Breast Cancer: After First Year, Females	Point Estimate	$4,921.18		11
Breast Cancer: Last Year, Females	Point Estimate	$88,699.10		11
Cervical Cancer: First Year, Females	Point Estimate	$66,084.92		11
Cervical Cancer: After First Year, Females	Point Estimate	$4,452.51		11
Cervical Cancer: Last Year, Females	Point Estimate	$109,789.89		11
Colorectal Cancer, First Year	Point Estimate	$75,341.45		11
Colorectal Cancer: After First Year	Point Estimate	$7,264.60		11

© 2024 Tamura K et al. *JAMA Network Open.*

Colorectal Cancer: Last Year	Point Estimate	$125,490.97		11
Esophageal Cancer, First Year	Point Estimate	$102,993.96		11
Esophageal Cancer: After First Year	Point Estimate	$11,951.44		11
Esophageal Cancer: Last Year	Point Estimate	$136,153.70		11
Renal Cancer: First Year	Point Estimate	$48,860.58		11
Renal Cancer: After First Year	Point Estimate	$11,717.22		11
Renal Cancer: Last Year	Point Estimate	$121,272.79		11
Pancreatic Cancer: First Year	Point Estimate	$127,951.55		11
Pancreatic Cancer: After First Year	Point Estimate	$25,074.76		11
Pancreatic Cancer: Last Year	Point Estimate	$147,284.87		11
Stomach Cancer: First Year	Point Estimate	$92,917.28		11
Stomach Cancer: After First Year	Point Estimate	$10,779.78		11
Stomach Cancer: Last Year	Point Estimate	$139,317.29		11
Uterine Cancer: First Year, Females	Point Estimate	$44,056.58		11
Uterine Cancer: After First Year, Females	Point Estimate	$3,398.02		11
Uterine Cancer: Last Year, Females	Point Estimate	$106,626.31		11
Prostate Cancer - First Year, Males	Point Estimate	$31,987.87		11
Prostate Cancer - After First Year, Males	Point Estimate	$3,280.75		11
Prostate Cancer - Last Year, Males	Point Estimate	$90,105.08		11
Annual Wages	Triangular	$65,669.71	$29,005 - $11,7007	12
**Probability Values**	**Distribution type**	**Mean**	**Range or standard deviation**	**Source**
**Probability of developing coronary heart disease (CHD)**				
At Chronic Health State 2 – non-smoker	Triangular	0.008	0 - 0.0250	13,14
At Chronic Health State 2 – smoker	Triangular	0.0124	0 – 0.04	13,14
At Chronic Health State 3 – non-smoker	Triangular	0.0094	0 - 0.0290	13,14
At Chronic Health State 3 – smoker	Triangular	0.01445	0 – 0.047	13,14
At Chronic Health State 4 – non-smoker	Triangular	0.0147	0 - 0.0430	13,14
At Chronic Health State 4 – smoker	Triangular	0.0216	0 - 0.063	13,14
Multiplier for increased probability of CHD for individual with overweight	Triangular	1.31	1.22 - 1.4	14
Multiplier for increased probability of CHD for individual with obesity	Triangular	1.56	1.54 - 1.58	14
**Probability of reoccurrence of CHD**				

At Chronic Health State 2	Triangular	0.0427	0 - 0.1	[15]
At Chronic Health State 3	Triangular	0.0347	0 - 0.057	[15]
At Chronic Health State 4	Triangular	0.0397	0 - 0.074	[15]
**Probability of death due to CHD**				
At Chronic Health State 2 – non-smoker	Triangular	0.0074	0.0003 - 0.0206	[13,14]
At Chronic Health State 2 – smoker	Triangular	0.0124	0.03277 – 0.0202	[13,14]
At Chronic Health State 3 – non-smoker	Triangular	0.0113	0.00035 - 0.0397	[13,14]
At Chronic Health State 3 – smoker	Triangular	0.01914	0.0040 – 0.0389	[13,14]
At Chronic Health State 4 – non-smoker	Triangular	0.0266	0.0007 - 0.113	[13,14]
At Chronic Health State 4 – smoker	Triangular	0.0477	0.008 – 0.111	[13,14]
Multiplier for increased probability of death for individual with overweight	Triangular	1.175	0.98 - 1.37	[13,14]
Multiplier for increased probability of death for individual with obesity	Triangular	1.375	1.3 - 1.45	[13,14]
**Probability of developing Stroke**				
At Chronic Health State 0 – non-smoker	Uniform	0.0025	0 - 0.007	[13,14,16]
At Chronic Health State 0 – smoker	Uniform	0.0042	0 – 0.012	[13,14,16]
At Chronic Health State 1 – non-smoker	Uniform	0.0033	0 - 0.009	[13,14,16]
At Chronic Health State 1 – smoker	Uniform	0.0056	0 – 0.015	[13,14,16]
At Chronic Health State 2 – non-smoker	Uniform	0.0038	0 - 0.01	[13,14,16]
At Chronic Health State 2 – smoker	Uniform	0.0063	0 – 0.017	[13,14,16]
At Chronic Health State 3 – non-smoker	Uniform	0.0037	0 - 0.01	[13,14,16]
At Chronic Health State 3 – smoker	Uniform	0.0062	0 – 0.016	[13,14,16]
At Chronic Health State 4 – non-smoker	Uniform	0.0070	0 - 0.017	[13,14,16]
At Chronic Health State 4 – smoker	Uniform	0.012	0 – 0.028	[13,14,16]
Multiplier for increased probability of stroke for individual with overweight	Uniform	1.115	1.06 - 1.17	[17,18]
Multiplier for increased probability of stroke for individual with obesity	Uniform	1.325	1.23 - 1.42	[17,18]
Probability of reoccurrence of Stroke at first year	Uniform	0.1287	0.0924 - 0.165	[19]
Probability of reoccurrence of Stroke after first year	Uniform	0.0443	0.0318 - 0.0567	[19]
**Probability of death due to stroke**				
In the first year of developing stroke	Uniform	0.188	0.135 - 0.241	[19]
After First year of developing stroke	Uniform	0.083	0.0596 - 0.1064	[19]
**Probabilities associated with diabetes and its associated complications[1]**				
Probability of developing T2DM	Point Estimate	0.25		[20,21]
Probability of developing Diabetic nephropathy by years of having T2DM	Uniform	0.14	0 - 0.28	[22]
Probability of developing Diabetic neuropathy by years of having T2DM	Uniform	0.36	0 - 0.72	[22]
Probability of Diabetic neuropathy by years of having T2DM	Uniform	0.14	0 - 0.28	[22]
Probability of developing blindness	Uniform	0.4	0 - 0.8	[23,24]
Probability of developing ESRD from Diabetic nephropathy	Point Estimate	0.0082		[25]
Probability of death from ESRD	Uniform	0.16	0.081 - 0.239	[26]
**Probabilities of developing Cancer for females**				
Breast	Uniform	0.0096	0 - 0.0191	[27,28]
Cervical	Uniform	0.00035	0 - 0.0007	[27,28]
Colorectal	Uniform	0.0033	0 - 0.0066	[27,28]
Esophageal	Uniform	0.0002	0 - 0.0004	[27,28]

© 2024 Tamura K et al. *JAMA Network Open.*

Renal	Uniform	0.0009	0 - 0.0018	27,28
Pancreatic	Uniform	0.00125	0 - 0.0025	27,28
Stomach	Uniform	0.0005	0 - 0.001	27,28
Uterine	Uniform	0.0022	0 - 0.0044	27,28
Multiplier for increased probability of developing cancer for females with overweight	Point Estimate	1.538		29
Multiplier for increased probability of developing cancer for females with obesity	Point Estimate	2.244		29
**Probabilities of developing Cancer for males**				
Colorectal	Uniform	0.0036	0 - 0.0071	27,28
Esophageal	Uniform	0.00065	0 - 0.0013	27,28
Renal	Uniform	0.00155	0 - 0.0031	27,28
Pancreatic	Uniform	0.00125	0 - 0.0025	27,28
Prostate	Uniform	0.0142	0 - 0.0284	27,28
Stomach	Uniform	0.00085	0 - 0.0017	27,28
Multiplier for increased probability of developing cancer for males with overweight	Point Estimate	1.55		29
Multiplier for increased probability of developing cancer for males with obesity	Point Estimate	2.243		29
**Probabilities of death due to Cancer for females**				
Breast	Uniform	0.034	0.008 - 0.06	28
Cervical	Uniform	0.0445	0.006 - 0.083	28
Colorectal	Uniform	0.066	0.027 - 0.105	28
Esophageal	Uniform	0.2305	0.035 - 0.426	28
Renal	Uniform	0.034	0.011 - 0.057	28
Pancreatic	Uniform	0.161	0.033 - 0.289	28
Stomach	Uniform	0.172	0.03 - 0.314	28
Uterine	Uniform	0.0275	0.007 - 0.048	28
Multiplier for increased probability of death from cancer for females with overweight	Point Estimate	1.14		30
Multiplier for increased probability of death from cancer for females with obesity	Point Estimate	1.41		30
**Probabilities of death due to Cancer for males**				
Colorectal	Uniform	0.089	0.016 - 0.162	
Esophageal	Uniform	0.2135	0.018 - 0.409	28
Renal	Uniform	0.0385	0.008 - 0.069	28
Pancreatic	Uniform	0.1765	0.018 - 0.335	28
Prostate	Uniform	0.033	0.015 - 0.051	28
Stomach	Uniform	0.1395	0.015 - 0.264	28
Multiplier for increased probability of death from cancer for males with overweight	Point Estimate	1.12		30
Multiplier for increased probability of death from cancer for males with obesity	Point Estimate	1.37		30
**Other Probabilities**				
Probability of death from all other causes for males	Uniform	0.000315 – 0.367		20,21
Probability of death from all other causes for females	Uniform	0.000788 – 0.426		20,21

© 2024 Tamura K et al. *JAMA Network Open.*

Probability of smoking for females	Uniform	0.3805	0.2652 – 0.4949	[20]
Probability of smoking for males	Uniform	0.4151	0.3302 – 0.5000	[20]
**Utility Values**	**Distribution type**	**Mean**	**Range or standard deviation**	**Source**
Stroke	Beta	0.6	0.09	[31-77]
CHD	Beta	0.73	0.1	[31-77]
Diabetic nephropathy	Beta	0.74	0.09	[31-77]
Diabetic neuropathy	Beta	0.65	0.04	[31-77]
Diabetic retinopathy	Beta	0.78	0.04	[31-77]
ESRD	Beta	0.63	0.03	[31-77]
Blindness	Beta	0.52	0.06	[31-77]
Renal Cancer	Beta	0.7	0.06	[31-77]
Cervical Cancer	Beta	0.63	0.11	[31-77]
Pancreatic Cancer	Beta	0.66	0.08	[31-77]
Gastric Cancer	Beta	0.52	0.08	[31-77]
Hypertension	Beta	0.97	0.01	[31-77]
Prostate Cancer	Beta	0.71	0.16	[31-77]
T2DM	Beta	0.85	0.08	[31-77]
Breast Cancer, First Year	Beta	0.66	0.06	[31-77]
Breast Cancer, After First Year	Beta	0.77	0.06	[31-77]
Breast Cancer, Last Year	Beta	0.23	0.001	[31-77]
Colon Cancer, First Year	Beta	0.52	0.12	[31-77]
Colon Cancer, After First Year	Beta	0.83	0.05	[31-77]
Colon Cancer, Last Year	Beta	0.3	0.001	[31-77]
Esophageal Cancer, First Year	Beta	0.71	0.22	[31-77]
Esophageal Cancer, After First Year	Beta	0.71	0.22	[31-77]
Esophageal Cancer, Last Year	Beta	0.34	0.001	[31-77]
Uterine Cancer, First Year	Beta	0.69	0.15	[31-77]
Uterine Cancer, After First Year	Beta	0.79	0.11	[31-77]
Uterine Cancer, Last Year	Beta	0.79	0.11	[31-77]

Note: CHD=Coronary Heart Disease; CHS = Chronic Health State; CVD = Cerebrovascular Disease; T2DM = Type 2 Diabetes Mellitus; ESRD = End-Stage Renal Disease

[1]The probabilities of developing T2DM are impacted by overweight and obesity but are accounted for in the values used for transition probabilities to move to Chronic Health State 3 or 4 for individuals with overweight or obesity (eTable 2 and eTable 3) and thus are not a separate weight-associated multiplier parameter.

© 2024 Tamura K et al. *JAMA Network Open.*

eTable 2. Transition probabilities for adults Age <= 45 years old[1]

	N_CHS0	N_CHS1	N_CHS2	N_CHS3	N_CHS4	OW_CHS0	OW_CHS1	OW_CHS2	OW_CHS3	OW_CHS4	OB_CHS0	OB_CHS1	OB_CHS2	OB_CHS3	OB_CHS4
**N_CHS0**	0.932	0.016	0.001	0	0	0.048	0.002	0	0	0	0	0	0	0	0
**N_CHS1**	0.023	0.9	0.007	0.007	0	0.002	0.051	0.002	0.002	0	0.001	0.001	0.003	0	0
**N_CHS2**	0.002	0.015	0.811	0.012	0.007	0	0	0.067	0.046	0.007	0	0	0.023	0.002	0.001
**N_CHS3**	0	0	0.024	0.844	0.002	0	0	0.046	0.067	0.003	0	0	0	0.002	0.001
**N_CHS4**	0	0	0	0.058	0.86	0	0	0	0	0.046	0	0	0	0	0.026
**OW_CHS0**	0.008	0.001	0	0	0	0.93	0.019	0.001	0	0	0.028	0.003	0.001	0	0
**OW_CHS1**	0.001	0.009	0.003	0.002	0.001	0.018	0.90	0.014	0.008	0	0.004	0.027	0.003	0.003	0
**OW_CHS2**	0	0.003	0.019	0.007	0	0.027	0.049	0.807	0.012	0.011	0	0.004	0.039	0.008	0.004
**OW_CHS3**	0	0	0	0.009	0	0	0	0.025	0.879	0	0	0	0.041	0.041	0
**OW_CHS4**	0	0	0	0.002	0.022	0	0	0	0.005	0.936	0	0	0	0	0.03
**OB_CHS0**	0	0	0	0	0	0.006	0	0	0	0	0.94	0.04	0.002	0.001	0.001
**OB_CHS1**	0	0.001	0	0	0	0.002	0.002	0	0	0	0.016	0.94	0.025	0.008	0.002
**OB_CHS2**	0	0.002	0	0	0	0	0.008	0.002	0.001	0	0.025	0.037	0.88	0.028	0.015
**OB_CHS3**	0	0	0.001	0.006	0	0	0	0.004	0.01	0.003	0	0	0.046	0.921	0.008
**OB_CHS4**	0	0	0	0	0.001	0	0	0	0	0.012	0	0	0	0	0.986

Note: N = Normal Weight; OW = Overweight; OB = Obesity

1: The transition probabilities were developed from data from multiple nationally representative studies. We obtained state transition probabilities from Coronary Artery Disease Risk Development in Young Adults (CARDIA)[20] and from the Atherosclerosis Risk in Communities (ARIC)[21] studies. These studies included diverse participants with different socioeconomic statuses, ages, genetic backgrounds, and environments.

© 2024 Tamura K et al. *JAMA Network Open.*

eTable 3. Transition probabilities for adults Age > 45 years old[1]

	N_CH S0	N_CH S1	N_CH S2	N_CH S3	N_CH S4	OW_CH S0	OW_CH S1	OW_CH S2	OW_CH S3	OW_CH S4	OB_CH S0	OB_CH S1	OB_CH S2	OB_CH S3	OB_CH S4
**N_CHS0**	0.865	0.102	0.001	0	0	0.012	0.011	0.002	0	0	0.001	0.001	0	0	0
**N_CHS1**	0.034	0.902	0.024	0.014	0	0.006	0.012	0.002	0.002	0	0.001	0	0.002	0	0
**N_CHS2**	0.036	0.053	0.869	0.015	0.004	0	0	0.015	0.002	0	0	0	0	0.001	0
**N_CHS3**	0	0	0.008	0.959	0.008	0	0	0	0.018	0.001	0	0	0	0	0
**N_CHS4**	0	0	0.007	0.011	0.948	0	0	0	0	0.018	0	0	0	0	0.002
**OW_CH S0**	0.008	0.004	0	0	0	0.876	0.069	0.013	0	0	0.013	0.014	0.001	0	0
**OW_CH S1**	0.004	0.004	0.003	0	0	0.023	0.912	0.027	0.014	0	0.002	0.007	0.002	0	0
**OW_CH S2**	0	0.007	0.004	0.001	0	0.003	0.039	0.90	0.019	0.006	0	0.002	0.009	0.001	0.001
**OW_CH S3**	0	0	0	0.005	0	0	0	0.013	0.957	0.01	0	0	0.001	0.009	0
**OW_CH S4**	0	0	0	0.001	0.007	0	0	0	0.009	0.962	0	0	0	0	0.01
**OB_CH S0**	0.001	0	0	0	0	0.007	0	0	0	0	0.866	0.079	0.043	0	0.002
**OB_CH S1**	0	0	0	0	0	0.003	0.005	0	0	0	0.016	0.913	0.039	0.02	0
**OB_CH S2**	0	0.001	0	0	0	0	0.001	0.005	0.001	0	0.001	0.032	0.919	0.026	0.011
**OB_CH S3**	0	0	0	0	0	0	0	0.001	0.006	0.001	0	0	0.013	0.959	0.017
**OB_CH S4**	0	0	0	0	0	0	0	0	0	0.008	0	0	0	0	0.984

Note: N = Normal Weight; OW = Overweight; OB = Obesity

1: The transition probabilities were developed from data from multiple nationally representative studies. We obtained state transition probabilities from Coronary Artery Disease Risk Development in Young Adults (CARDIA)[20] and from the Atherosclerosis Risk in Communities (ARIC)[21] studies. These studies included diverse participants with different socioeconomic statuses, ages, genetic backgrounds, and environments.

© 2024 Tamura K et al. *JAMA Network Open.*

eReferences.

1. Ferguson MC, Bartsch SM, O'Shea KJ, et al. The potential epidemiologic, clinical, and economic impact of requiring schools to offer Physical Education (PE) classes in Mexico City. *PLoS One.* 2022;17(5):e0268118.
2. Lee BY, Adam A, Zenkov E, et al. Modeling The Economic And Health Impact Of Increasing Children's Physical Activity In The United States. *Health Aff (Millwood).* 2017;36(5):902-908.
3. Fallah-Fini S, Adam A, Cheskin LJ, Bartsch SM, Lee BY. The Additional Costs and Health Effects of a Patient Having Overweight or Obesity: A Computational Model. *Obesity (Silver Spring).* 2017;25(10):1809-1815.
4. Ferguson MC, Morgan MJ, O'Shea KJ, et al. Using Simulation Modeling to Guide the Design of the Girl Scouts Fierce & Fit Program. *Obesity (Silver Spring).* 2020;28(7):1317-1324.
5. Agency for Healthcare Research and Quality. Medical Expenditure Panel Survey In: Agency for Healthcare Research and Quality, ed: Agency for Healthcare Research and Quality,; 2012.
6. Medical Expenditure Panel Survey (MEPS) Household Component (HC) 2020. https://datatools.ahrq.gov/meps-hc?type=tab&tab=mepshch3uep.
7. Nichols GA, Vupputuri S, Lau H. Medical care costs associated with progression of diabetic nephropathy. *Diabetes Care.* 2011;34(11):2374-2378.
8. United States Renal Data System. *2022 USRDS Annual Data Report: Epidemiology of kidney disease in the United States.* National Institutes of Health, National Institute of Diabetes and Digestive and Kidney Diseases, Bethesda, MD, 2022.
9. Sadosky A, Mardekian J, Parsons B, Hopps M, Bienen EJ, Markman J. Healthcare utilization and costs in diabetes relative to the clinical spectrum of painful diabetic peripheral neuropathy. *J Diabetes Complications.* 2015;29(2):212-217.
10. NORC at the University of Chicago. *Cost of Vision Problems: The Economic Burden of Vision Loss and Eye Disorders in the United States.* 2013.
11. National Cancer Institute N, HHS, . *Cancer Trends Progress Report.* 2022.
12. Bureau of Labor Statistics U.S. Department of Labor. Occupational Employment and Wage Statistics. Bureau of Labor Statistics. http://www.bls.gov/oes/current/oes_nat.htm. Published 2022. Updated May 2022. Accessed 05/18/2023, 2023.
13. D'Agostino Sr RB, Grundy S, Sullivan LM, Wilson P. Validation of the Framingham coronary heart disease prediction scores: results of a multiple ethnic groups investigation. *Jama.* 2001;286(2):180-187.
14. Wilson PW, D'Agostino RB, Sullivan L, Parise H, Kannel WB. Overweight and obesity as determinants of cardiovascular risk: the Framingham experience. *Archives of internal medicine.* 2002;162(16):1867-1872.
15. Rea TD, Heckbert SR, Kaplan RC, Smith NL, Lemaitre RN, Psaty BM. Smoking status and risk for recurrent coronary events after myocardial infarction. *Annals of Internal Medicine.* 2002;137(6):494-500.
16. Pender JR, Pories WJ. Epidemiology of obesity in the United States. *Gastroenterology Clinics of North America.* 2005;34(1):1-7.
17. Rexrode KM, Hennekens CH, Willett WC, et al. A prospective study of body mass index, weight change, and risk of stroke in women. *Jama.* 1997;277(19):1539-1545.
18. Walker SP, Rimm EB, Ascherio A, Kawachi I, Stampfer MJ, Willett WC. Body size and fat distribution as predictors of stroke among US men. *American Journal of Epidemiology.* 1996;144(12):1143-1150.

© 2024 Tamura K et al. *JAMA Network Open.*

19. Sacco RL, Shi T, Zamanillo M, Kargman D. Predictors of mortality and recurrence after hospitalized cerebral infarction in an urban community The Northern Manhattan Stroke Study. *Neurology.* 1994;44(4):626-626.
20. Friedman GD, Cutter GR, Donahue RP, et al. CARDIA: study design, recruitment, and some characteristics of the examined subjects. *J Clin Epidemiol.* 1988;41(11):1105-1116.
21. The ARIC Investigators. The Atherosclerosis Risk in Community Study: Design and Objectives. *American Journal of Epidemiology.* 1989;129(4):687-702.
22. Orchard TJ, Dorman JS, Maser RE, et al. Prevalence of complications in IDDM by sex and duration: Pittsburgh Epidemiology of Diabetes Complications Study II. *Diabetes.* 1990;39(9):1116-1124.
23. Yau JW, Rogers SL, Kawasaki R, et al. Global prevalence and major risk factors of diabetic retinopathy. *Diabetes care.* 2012;35(3):556-564.
24. Klein R, Klein BE, Moss SE, Cruickshanks KJ. Relationship of hyperglycemia to the long-term incidence and progression of diabetic retinopathy. *Archives of internal medicine.* 1994;154(19):2169-2178.
25. Kiberd BA, Clase CM. Cumulative risk for developing end-stage renal disease in the US population. *Journal of the American Society of Nephrology.* 2002;13(6):1635-1644.
26. Saran R, Li Y, Robinson B, et al. US Renal Data System 2015 Annual Data Report: Epidemiology of Kidney Disease in the United States. *American journal of kidney diseases: the official journal of the National Kidney Foundation.* 2016;67(3 Suppl 1):A7.
27. Fay MP, Pfeiffer R, Cronin KA, Le C, Feuer EJ. Age-conditional probabilities of developing cancer. *Statistics in medicine.* 2003;22(11):1837-1848.
28. Hayat MJ, Howlader N, Reichman ME, Edwards BK. Cancer statistics, trends, and multiple primary cancer analyses from the Surveillance, Epidemiology, and End Results (SEER) Program. *The oncologist.* 2007;12(1):20-37.
29. Calle EE, Kaaks R. Overweight, obesity and cancer: epidemiological evidence and proposed mechanisms. *Nat Rev Cancer.* 2004;4(8):579-591.
30. Patel AV, Hildebrand JS, Gapstur SM. Body Mass Index and All-Cause Mortality in a Large Prospective Cohort of White and Black U.S. Adults. *PLOS ONE.* 2014;9(10):e109153.
31. Adarkwah CC, Gandjour A, Akkerman M, Evers S. To treat or not to treat? Cost-effectiveness of ace inhibitors in non-diabetic advanced renal disease - a Dutch perspective. *Kidney Blood Press Res.* 2013;37(2-3):168-180.
32. Aspinall SL, Smith KJ, Good CB, et al. Incremental cost effectiveness of pharmacist-managed erythropoiesis-stimulating agent clinics for non-dialysis-dependent chronic kidney disease patients. *Appl Health Econ Health Policy.* 2013;11(6):653-660.
33. Athanasakis K, Petrakis I, Karampli E, Vitsou E, Lyras L, Kyriopoulos J. Pregabalin versus gabapentin in the management of peripheral neuropathic pain associated with post-herpetic neuralgia and diabetic neuropathy: a cost effectiveness analysis for the Greek healthcare setting. *BMC Neurol.* 2013;13:56.
34. Attard CL, Brown S, Alloul K, Moore MJ. Cost-effectiveness of folfirinox for first-line treatment of metastatic pancreatic cancer. *Curr Oncol.* 2014;21(1):e41-51.
35. Bellows BK, Dahal A, Jiao T, Biskupiak J. A cost-utility analysis of pregabalin versus duloxetine for the treatment of painful diabetic neuropathy. *J Pain Palliat Care Pharmacother.* 2012;26(2):153-164.
36. Boger PC, Turner D, Roderick P, Patel P. A UK-based cost-utility analysis of radiofrequency ablation or oesophagectomy for the management of high-grade dysplasia in Barrett's oesophagus. *Aliment Pharmacol Ther.* 2010;32(11-12):1332-1342.

© 2024 Tamura K et al. *JAMA Network Open.*

37. Borisenko O, Beige J, Lovett EG, Hoppe UC, Bjessmo S. Cost-effectiveness of Barostim therapy for the treatment of resistant hypertension in European settings. *J Hypertens.* 2014;32(3):681-692.
38. Botteman MF, Meijboom M, Foley I, Stephens JM, Chen YM, Kaura S. Cost-effectiveness of zoledronic acid in the prevention of skeletal-related events in patients with bone metastases secondary to advanced renal cell carcinoma: application to France, Germany, and the United Kingdom. *Eur J Health Econ.* 2011;12(6):575-588.
39. Bresse X, Goergen C, Prager B, Joura E. Universal vaccination with the quadrivalent HPV vaccine in Austria: impact on virus circulation, public health and cost-effectiveness analysis. *Expert Rev Pharmacoecon Outcomes Res.* 2014;14(2):269-281.
40. Brown ST, Grima DG, Sauriol L. Cost-effectiveness of insulin glargine versus sitagliptin in insulin-naive patients with type 2 diabetes mellitus. *Clin Ther.* 2014;36(11):1576-1587.
41. Choudhry NK, Patrick AR, Glynn RJ, Avorn J. The cost-effectiveness of C-reactive protein testing and rosuvastatin treatment for patients with normal cholesterol levels. *J Am Coll Cardiol.* 2011;57(7):784-791.
42. Das A, Ngamruengphong S, Nagendra S, Chak A. Asymptomatic pancreatic cystic neoplasm: a cost-effectiveness analysis of different strategies of management. *Gastrointest Endosc.* 2009;70(4):690-699 e696.
43. Dorian P, Kongnakorn T, Phatak H, et al. Cost-effectiveness of apixaban vs. current standard of care for stroke prevention in patients with atrial fibrillation. *Eur Heart J.* 2014;35(28):1897-1906.
44. Folse HJ, Goswami D, Rengarajan B, Budoff M, Kahn R. Clinical- and cost-effectiveness of LDL particle-guided statin therapy: a simulation study. *Atherosclerosis.* 2014;236(1):154-161.
45. Fonseca T, Clegg J, Caputo G, Norrbacka K, Dilla T, Alvarez M. The cost-effectiveness of exenatide once weekly compared with exenatide twice daily and insulin glargine for the treatment of patients with type two diabetes and body mass index >/=30 kg/m(2) in Spain. *J Med Econ.* 2013;16(7):926-938.
46. Gladwell D, Henry T, Cook M, Akehurst R. Cost effectiveness of renal denervation therapy for the treatment of resistant hypertension in the UK. *Appl Health Econ Health Policy.* 2014;12(6):611-622.
47. Gomes M, Aldridge RW, Wylie P, Bell J, Epstein O. Cost-effectiveness analysis of 3-D computerized tomography colonography versus optical colonoscopy for imaging symptomatic gastroenterology patients. *Appl Health Econ Health Policy.* 2013;11(2):107-117.
48. Green LE, Dinh TA, Hinds DA, Walser BL, Allman R. Economic evaluation of using a genetic test to direct breast cancer chemoprevention in white women with a previous breast biopsy. *Appl Health Econ Health Policy.* 2014;12(2):203-217.
49. Hoyle M, Green C, Thompson-Coon J, et al. Cost-effectiveness of temsirolimus for first line treatment of advanced renal cell carcinoma. *Value Health.* 2010;13(1):61-68.
50. Kaambwa B, Bryan S, Jowett S, et al. Telemonitoring and self-management in the control of hypertension (TASMINH2): a cost-effectiveness analysis. *Eur J Prev Cardiol.* 2014;21(12):1517-1530.
51. Kohn CG, Parker MW, Limone BL, Coleman CI. Cost-effectiveness of ranolazine added to standard-of-care treatment in patients with chronic stable angina pectoris. *Am J Cardiol.* 2014;113(8):1306-1311.
52. Kondo M, Yamagata K, Hoshi SL, et al. Cost-effectiveness of chronic kidney disease mass screening test in Japan. *Clin Exp Nephrol.* 2012;16(2):279-291.
53. Kongnakorn T, Lanitis T, Annemans L, et al. Stroke and systemic embolism prevention in patients with atrial fibrillation in Belgium: comparative cost effectiveness of new oral anticoagulants and warfarin. *Clin Drug Investig.* 2015;35(2):109-119.

© 2024 Tamura K et al. *JAMA Network Open.*

54. Kreidieh B, Manero MR, Cortez SH, Schurmann P, Valderrabano M. The Cost Effectiveness of LAA Exclusion. *J Atr Fibrillation.* 2016;8(5):1374.
55. Krzyzanowska MK, Earle CC, Kuntz KM, Weeks JC. Using economic analysis to evaluate the potential of multimodality therapy for elderly patients with locally advanced pancreatic cancer. *Int J Radiat Oncol Biol Phys.* 2007;67(1):211-218.
56. Kwon JS, Carey MS, Goldie SJ, Kim JJ. Cost-effectiveness analysis of treatment strategies for Stage I and II endometrial cancer. *J Obstet Gynaecol Can.* 2007;29(2):131-139.
57. Lairson DR, Parikh RC, Cormier JN, Chan W, Du XL. Cost-utility analysis of chemotherapy regimens in elderly patients with stage III colon cancer. *Pharmacoeconomics.* 2014;32(10):1005-1013.
58. Lee CI, Cevik M, Alagoz O, et al. Comparative effectiveness of combined digital mammography and tomosynthesis screening for women with dense breasts. *Radiology.* 2015;274(3):772-780.
59. Masterton RG, Casamayor M, Musingarimi P, et al. De-escalation from micafungin is a cost-effective alternative to traditional escalation from fluconazole in the treatment of patients with systemic Candida infections. *J Med Econ.* 2013;16(11):1344-1356.
60. Melnikow J, Birch S, Slee C, McCarthy TJ, Helms LJ, Kuppermann M. Tamoxifen for breast cancer risk reduction: impact of alternative approaches to quality-of-life adjustment on cost-effectiveness analysis. *Med Care.* 2008;46(9):946-953.
61. Morais J, Aguiar C, McLeod E, Chatzitheofilou I, Fonseca Santos I, Pereira S. Cost-effectiveness of rivaroxaban for stroke prevention in atrial fibrillation in the Portuguese setting. *Rev Port Cardiol.* 2014;33(9):535-544.
62. Murphy JD, Chang DT, Abelson J, et al. Cost-effectiveness of modern radiotherapy techniques in locally advanced pancreatic cancer. *Cancer.* 2012;118(4):1119-1129.
63. Pataky R, Ismail Z, Coldman AJ, et al. Cost-effectiveness of annual versus biennial screening mammography for women with high mammographic breast density. *J Med Screen.* 2014;21(4):180-188.
64. Paz-Ares L, del Muro JG, Grande E, Diaz S. A cost-effectiveness analysis of sunitinib in patients with metastatic renal cell carcinoma intolerant to or experiencing disease progression on immunotherapy: perspective of the Spanish National Health System. *J Clin Pharm Ther.* 2010;35(4):429-438.
65. Pietzsch JB, Liu S, Garner AM, Kezirian EJ, Strollo PJ. Long-Term Cost-Effectiveness of Upper Airway Stimulation for the Treatment of Obstructive Sleep Apnea: A Model-Based Projection Based on the STAR Trial. *Sleep.* 2015;38(5):735-744.
66. Rachapelle S, Legood R, Alavi Y, et al. The cost-utility of telemedicine to screen for diabetic retinopathy in India. *Ophthalmology.* 2013;120(3):566-573.
67. Rupnow MF, Chang AH, Shachter RD, Owens DK, Parsonnet J. Cost-effectiveness of a potential prophylactic Helicobacter pylori vaccine in the United States. *J Infect Dis.* 2009;200(8):1311-1317.
68. Schaufler TM, Wolff M. Cost effectiveness of preventive screening programmes for type 2 diabetes mellitus in Germany. *Appl Health Econ Health Policy.* 2010;8(3):191-202.
69. Scotland GS, McNamee P, Fleming AD, et al. Costs and consequences of automated algorithms versus manual grading for the detection of referable diabetic retinopathy. *Br J Ophthalmol.* 2010;94(6):712-719.
70. Sharaiha RZ, Freedberg DE, Abrams JA, Wang YC. Cost-effectiveness of chemoprevention with proton pump inhibitors in Barrett's esophagus. *Dig Dis Sci.* 2014;59(6):1222-1230.
71. Wilson FA, Villarreal R, Stimpson JP, Pagan JA. Cost-effectiveness analysis of a colonoscopy screening navigator program designed for Hispanic men. *J Cancer Educ.* 2015;30(2):260-267.

© 2024 Tamura K et al. *JAMA Network Open.*

72. Wisloff T, Hagen G, Klemp M. Economic evaluation of warfarin, dabigatran, rivaroxaban, and apixaban for stroke prevention in atrial fibrillation. *Pharmacoeconomics.* 2014;32(6):601-612.
73. Liu M, Wu L, Ming Q. How Does Physical Activity Intervention Improve Self-Esteem and Self-Concept in Children and Adolescents? Evidence from a Meta-Analysis. *PLOS ONE.* 2015;10(8):e0134804.
74. Wu B, Kun L, Liu X, He B. Cost-effectiveness of different strategies for stroke prevention in patients with atrial fibrillation in a health resource-limited setting. *Cardiovasc Drugs Ther.* 2014;28(1):87-98.
75. Xie F, O'Reilly D, Ferrusi IL, et al. Illustrating economic evaluation of diagnostic technologies: comparing Helicobacter pylori screening strategies in prevention of gastric cancer in Canada. *J Am Coll Radiol.* 2009;6(5):317-323.
76. Yang KY, Caughey AB, Little SE, Cheung MK, Chen LM. A cost-effectiveness analysis of prophylactic surgery versus gynecologic surveillance for women from hereditary non-polyposis colorectal cancer (HNPCC) Families. *Fam Cancer.* 2011;10(3):535-543.
77. Zhou HJ, Dan YY, Naidoo N, Li SC, Yeoh KG. A cost-effectiveness analysis evaluating endoscopic surveillance for gastric cancer for populations with low to intermediate risk. *PLoS One.* 2013;8(12):e83959.

© 2024 Tamura K et al. *JAMA Network Open.*
