# Supplementary material for: Modeling Health and Economic Outcomes of Eliminating Sex Disparities in Youth Physical Activity
Source: JAMA Netw Open. 2024 Nov 25;7(11):e2446775. doi: 10.1001/jamanetworkopen.2024.46775 (PMC11589798; doi:10.1001/jamanetworkopen.2024.46775)
Supplement: Supplement 2. — Data Sharing Statement [file jamanetwopen-e2446775-s002.pdf]

Data Sharing Statement

Tamura. Modeling Health and Economic Outcomes of Eliminating Sex Disparities in Youth Physical Activity. *JAMA Netw Open*. Published November 25, 2024. doi:10.1001/jamanetworkopen.2024.46775

Data
Data available: Yes
Data types: Data (not involving human participants)
How to access data: All input data is included in the Methods and Supplemental File.
When available: With publication

Supporting Documents
Document types: None

Additional Information
Who can access the data: All input data is included in the Methods and Supplemental File.
Types of analyses: All input data is included in the Methods and Supplemental File.
Mechanisms of data availability: All input data is included in the Methods and Supplemental File.
